# Digital Health Data Quality Issues: Systematic Review

**DOI:** 10.2196/42615

**Published:** 2023-03-31

**Authors:** Rehan Syed, Rebekah Eden, Tendai Makasi, Ignatius Chukwudi, Azumah Mamudu, Mostafa Kamalpour, Dakshi Kapugama Geeganage, Sareh Sadeghianasl, Sander J J Leemans, Kanika Goel, Robert Andrews, Moe Thandar Wynn, Arthur ter Hofstede, Trina Myers

**Affiliations:** 1 School of Information Systems Faculty of Science Queensland University of Technology Brisbane Australia; 2 Rheinisch-Westfälische Technische Hochschule Aachen University Aachen Germany

**Keywords:** data quality, digital health, electronic health record, eHealth, systematic reviews

## Abstract

**Background:**

The promise of digital health is principally dependent on the ability to electronically capture data that can be analyzed to improve decision-making. However, the ability to effectively harness data has proven elusive, largely because of the quality of the data captured. Despite the importance of data quality (DQ), an agreed-upon DQ taxonomy evades literature. When consolidated frameworks are developed, the dimensions are often fragmented, without consideration of the interrelationships among the dimensions or their resultant impact.

**Objective:**

The aim of this study was to develop a consolidated digital health DQ dimension and outcome (DQ-DO) framework to provide insights into 3 research questions: What are the dimensions of digital health DQ? How are the dimensions of digital health DQ related? and What are the impacts of digital health DQ?

**Methods:**

Following the PRISMA (Preferred Reporting Items for Systematic Reviews and Meta-Analyses) guidelines, a developmental systematic literature review was conducted of peer-reviewed literature focusing on digital health DQ in predominately hospital settings. A total of 227 relevant articles were retrieved and inductively analyzed to identify digital health DQ dimensions and outcomes. The inductive analysis was performed through open coding, constant comparison, and card sorting with subject matter experts to identify digital health DQ dimensions and digital health DQ outcomes. Subsequently, a computer-assisted analysis was performed and verified by DQ experts to identify the interrelationships among the DQ dimensions and relationships between DQ dimensions and outcomes. The analysis resulted in the development of the DQ-DO framework.

**Results:**

The digital health DQ-DO framework consists of 6 dimensions of DQ, namely accessibility, accuracy, completeness, consistency, contextual validity, and currency; interrelationships among the dimensions of digital health DQ, with consistency being the most influential dimension impacting all other digital health DQ dimensions; 5 digital health DQ outcomes, namely clinical, clinician, research-related, business process, and organizational outcomes; and relationships between the digital health DQ dimensions and DQ outcomes, with the consistency and accessibility dimensions impacting all DQ outcomes.

**Conclusions:**

The DQ-DO framework developed in this study demonstrates the complexity of digital health DQ and the necessity for reducing digital health DQ issues. The framework further provides health care executives with holistic insights into DQ issues and resultant outcomes, which can help them prioritize which DQ-related problems to tackle first.

## Introduction

### Background

The health care landscape is changing globally owing to substantial investments in health information systems that seek to improve health care outcomes [1]. Despite the rapid adoption of health information systems [2] and the perception of digital health as a panacea [3] for improving health care quality, the outcomes have been mixed [4,5]. As Reisman [6] noted, despite substantial investment and effort and widespread application of digital health, many of the promised benefits have yet to be realized.

The promise of digital health is principally dependent on the ability to electronically capture data that can be analyzed to improve decision-making at the local, national [6], and global levels [7]. However, the ability to harness data effectively and meaningfully has proven difficult and elusive, largely because of the quality of the data captured. Darko-Yawson and Ellingsen [8] highlighted that digital health has resulted in more bad data rather than improving the quality of data. It is widely accepted that the data from digital health are plagued by accuracy and completeness concerns [9-12]. Poor data quality (DQ) can be detrimental to continuity of care [13], patient safety [14], clinician productivity [15], and research [16].

To assess DQ, scholars have developed numerous DQ taxonomies, which evaluate the extent to which the data contained within digital health systems adhere to multiple dimensions (ie, measurable components of DQ). Weiskopf and Weng [17] identified 5 dimensions of DQ spanning completeness, correctness, concordance, plausibility, and currency. Subsequently, Weiskopf et al [18] refined the typology to consist of only 3 dimensions: completeness, correctness, and currency. Similarly, Puttkammer et al [13] focused on completeness, accuracy, and timeliness, whereas Kahn et al [19] examined conformance, completeness, and plausibility. Others identified “fitness of use” [20] and the validity of data to a specific context [21] as key DQ dimensions. Overall, there are wide-ranging definitions of DQ, with an agreed-upon taxonomy evading the literature. In this paper, upon synthesizing the literature, we define DQ as the extent to which digital health data are accessible, accurate, complete, consistent, contextually valid, and current. When consolidated frameworks are developed, the dimensions are often treated in a fragmented manner, with few attempts to understand the relationships between the dimensions and the resultant outcomes. This is substantiated by Bettencourt-Silva et al [22], who indicated that DQ is not systematically or consistently assessed.

### Research Aims and Questions

Failure of health organizations to leverage high-quality data will compromise the sustainability of an already strained health care system [23]. Therefore, we undertook a systematic literature review to answer the following research questions: (1) What are the dimensions of digital health DQ? (2) How are the dimensions of digital health DQ related? and (3) What are the impacts of digital health DQ? The aim of this research was to develop, from synthesizing the literature, a consolidated digital health DQ dimension and outcome (DQ-DO) framework, which demonstrates the DQ dimensions and their interrelationships as well as their impact on core health care outcomes. The consolidated DQ-DO framework will be beneficial to both research and practice. For researchers, our review consolidates the digital health DQ literature and provides core areas for future research to rigorously evaluate and improve digital health DQ. For practice, this study provides health care executives and strategic decision makers with insights into both the criticality of digital health DQ by exemplifying the impacts and the complexity of digital health DQ by demonstrating the interrelationships between the dimensions. Multimedia Appendix 1 [24] provides a list of common acronyms used in this study.

This paper is structured as follows: first, we provide details of the systematic literature review method; second, in line with the research questions, we present our 3 key findings—(1) DQ dimensions, (2) DQ interrelationships, and (3) DQ outcomes; and third, we compare the findings of our study with those of previous studies and discuss the implications of this work.

## Methods

We followed the PRISMA (Preferred Reporting Items for Systematic Reviews and Meta-Analyses) guidelines and the guidelines proposed by Webster and Watson [25] for systematic literature reviews. Specifically, consistent with Templier and Paré [26], this systematic literature review was developmental in nature with the goal of developing a consolidated digital health DQ framework.

### Literature Search and Selection

To ensure the completeness of the review [25] and consistent with interdisciplinary reviews, the literature search spanned multiple fields and databases (ie, PubMed, Public Health, Cochrane, SpringerLink, EBSCOhost [MEDLINE and PsycInfo], ABI/INFORM, AISel, Emerald Insight, IEEE Xplore digital library, Scopus, and ACM Digital Library). The search was conducted in October 2021 and was not constrained by the year of publication because the concept of DQ has a long-standing academic history. The search terms were reflective of our research topic and research questions. To ensure comprehensiveness, the search terms were broadened by searching their synonyms. For example, we used search terms such as “electronic health record,” “digital health record,” “e-health,” “electronic medical record,” “EHR,” “EMR,” “data quality,” “data reduction,” “data cleaning,” “data pre-processing,” “information quality,” “data cleansing,” “data preparation,” “intelligence quality,” “data wrangling,” and “data transformation.” Keywords and search queries were reviewed by the reference librarian and subject matter experts in digital health (Multimedia Appendix 2).

**Figure 1:**
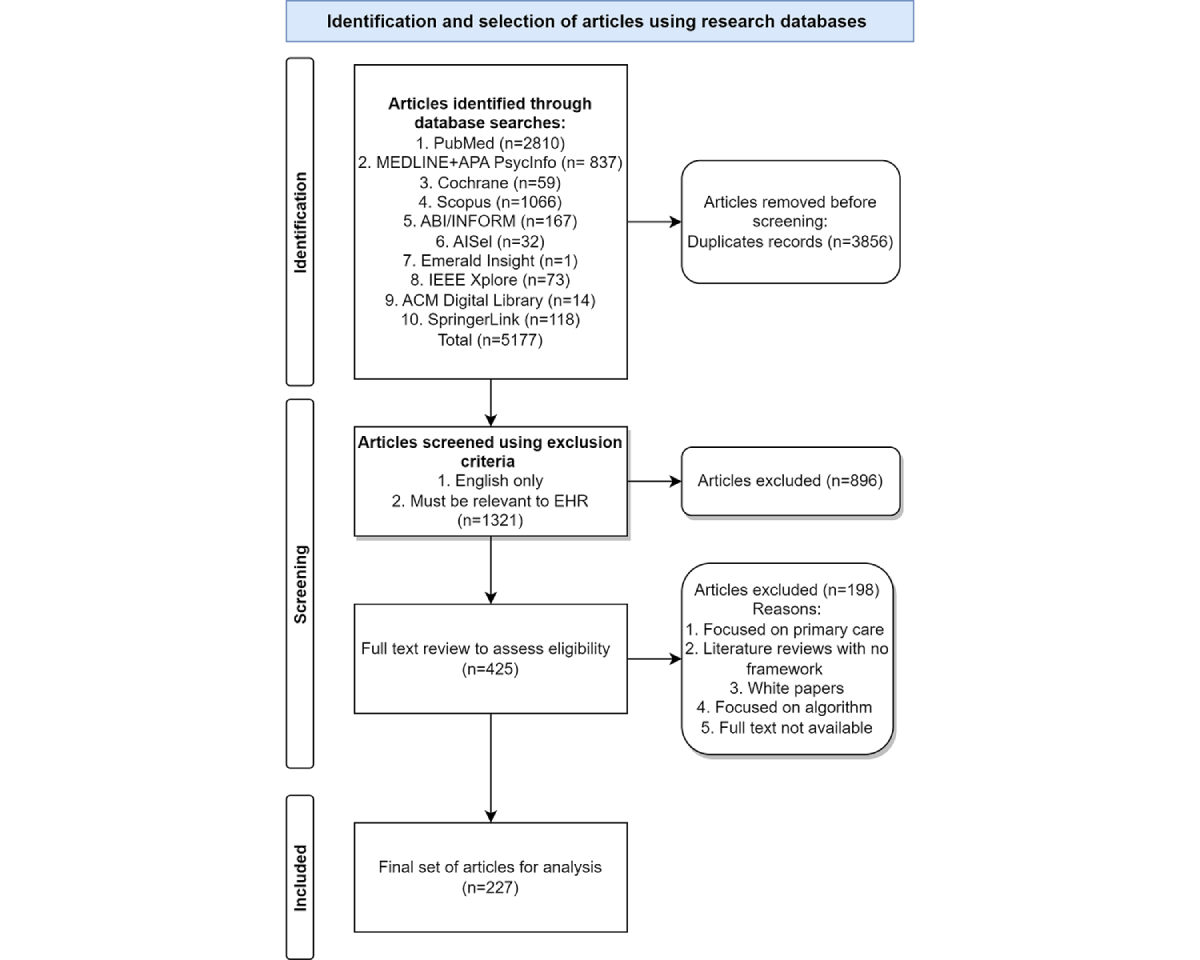
PRISMA (Preferred Reporting Items for Systematic Reviews and Meta-Analyses) inclusion process. EHR: electronic health record.

The papers returned from the search were narrowed down in a 4-step process (Figure 1). In the identification step, 5177 articles were identified through multiple database searches, and from these, 3856 (74.48%) duplicates were removed, resulting in 1321 (25.42%) articles. These 1321 articles were randomly divided into 6 batches, which were assigned to separate researchers, who applied the inclusion and exclusion criteria (Textbox 1). As a result of abstract screening, 67.83% (896/1321) of articles were excluded, resulting in 425 (32.17%) articles. Following an approach to the abstract screening, the 425 articles were again randomly divided into 6 batches and assigned to 1 of the 6 researchers to read and assess the relevance of the article in line with the selection criteria. The assessment of each of the 425 articles was then verified by the research team, resulting in a final set of 227 (53.4%) relevant articles. During this screening phase (ie, abstract and full text), daily meetings were held with the research team in which any uncertainties were raised and discussed until consensus was reached by the team as to whether the article should be included or excluded from the review In line with Templier and Paré [26], as this systematic literature review was developmental in nature rather than an aggregative meta-analysis, quality appraisals were not performed on individual articles.

**Inclusion and exclusion criteria.**

**Inclusion criteria**

- Specifically focuses on data quality in digital health
- Empirical papers or review articles where conceptual frameworks were either developed or assessed
- Considers digital health within hospital settings
- Published in peer-reviewed outlets within any time frame
- Published in English

**Exclusion criteria**

- Development of algorithms for advanced analytics techniques (eg, machine learning and artificial intelligence) without application within hospital settings
- Descriptive papers without a conceptual framework or an empirical analysis
- Focused only on primary care (eg, general practice)
- Pre–go-live considerations (eg, software development)
- Theses and non–peer-reviewed publications (eg, white papers and editorials)

### Literature Analysis

**Figure 2:**
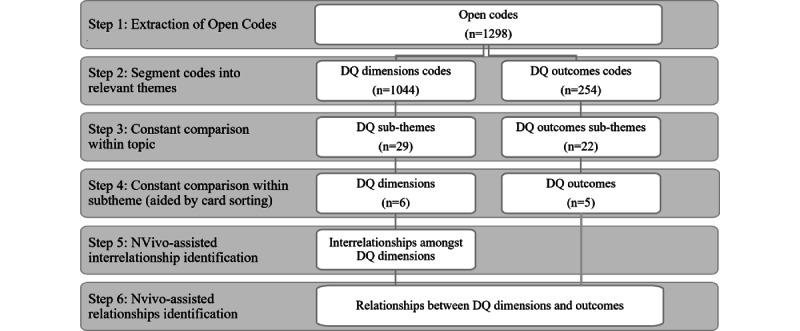
Analysis process. DQ: data quality.

The relevant articles were imported to NVivo (version 12; QSR International), where the analysis was iteratively performed. To ensure reliability and consistency in coding, a coding rule book [27] was developed and progressively updated to guide the coding process. The analysis involved 6 steps (Figure 2).

In the first step of the analysis, the research team performed open coding [27], where relevant statements from each article were extracted using verbatim codes and grouped based on similarities [28]. The first round of coding resulted in 1298 open codes. Second, the open codes were segmented into 2 high-level themes: the first group contained 1044 (80.43%) open codes pertaining directly to DQ dimensions (eg, data accuracy), and the second group contained 254 (19.57%) open codes pertaining to DQ outcomes (eg, financial outcomes).

In the third step, through constant comparison [29], the 1044 raw DQ codes were combined into 29 DQ subthemes based on commonalities (eg, contextual DQ, fitness for use, granularity, relevancy, accessibility, and availability). In the fourth step, again by performing iterative and multiple rounds of constant comparison, the 254 open codes related to DQ outcomes were used to construct 22 initial DQ outcome subthemes (eg, patient safety, clinician-patient relationship, and continuity of care). The DQ outcome subthemes were further compared with each other, resulting in 5 DQ outcome dimensions (eg, clinical, business process, research-related, clinician, and organizational outcomes). For the DQ subthemes, a constant comparison was performed using the card-sorting method [30], where an expert panel of 8 DQ researchers split into 4 groups assessed the subthemes for commonalities and differences. The expert groups presented their categorization to each other until a consensus was reached. This resulted in a consolidated set of 6 DQ dimensions (accuracy, consistency, completeness, contextual validity, accessibility, and currency). Multimedia Appendix 3 [9,12,13,15,16,18,19,21,31-65] provides an example of how the open codes were reflected in the subthemes and themes.

After identifying the DQ dimensions and outcomes, the next stage of coding progressed to identifying the interrelationships (step 5) among the DQ dimensions and the relationships (step 6) between the DQ dimensions and DQ outcomes. To this end, the matrix coding query function using relevant Boolean operators (AND and NEAR) in NVivo was performed. The outcomes of the matrix queries were reviewed and verified by an expert researcher in the health domain.

Throughout the analysis, steps for providing credibility to our findings were performed. First, before commencing the analysis, the research team members who extracted the verbatim codes initially independently reviewed 3 common articles and then convened to review any variations in coding. In addition, they reconvened multiple times a week to discuss their coding and update the codebook to ensure that a consistent approach was followed. Coder corroboration was performed throughout the analysis, with 2 experienced researchers independently verifying all verbatim codes until a consensus was reached [27]. Subsequent coder corroboration was performed by 2 experienced researchers to ensure that the open codes were accurately mapped to the themes and dimensions. This served to provide internal reliability. Steps for improving external reliability were also performed [66]. Specifically, the card-sorting method provided an expert appraisal. In addition, the findings were presented to and confirmed by 3 digital health care professionals.

## Results

### Overview

The vast majority of relevant articles were published in journal outlets (169/227, 74.4%), followed by conference proceedings (42/227, 18.5%) and book sections (16/227, 7%). The 169 journal articles were published in 107 journals, with 12% (n=13) of the journals publishing >1 study (these journals are *BMC Medical Informatics and Decision Making, eGEMS*, *International Journal of Medical Informatics*, *Applied Clinical Informatics*, *Journal of Medical Internet Research*, *Journal of the American Medical Information Association*, *PLOS One*, *BMC Emergency Medicine*, *Computer Methods and Programs in Biomedicine*, *International Journal of Population Data Science*, *JCO Clinical Cancer Informatics*, *Perspectives in Health Information Management*, *Studies in Health Technology and Informatics*, *Australian Health Review*, *MBC Health Services Research*, *BMJ Open*, *Decision Support Systems*, *Health Informatics Journal*, *International Journal of Information Management*, *JAMIA Open*, *JMIR Medical Informatics*, *Journal of Biomedical Informatics*, *Journal of Medical Systems*, *Malawi Medical Journal*, *Medical Care*, *Online Journal of Public Health Informatics*, and *Telemedicine and e-Health*). A complete breakdown of the number of articles published in each outlet is provided in Multimedia Appendix 4.

**Figure 3:**
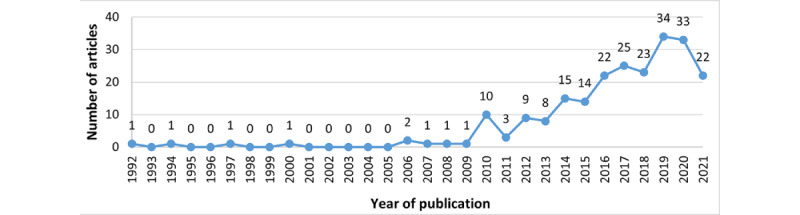
Publications by year.

Overall, as illustrated in Figure 3, the interest in digital health DQ has been increasing over time, with sporadic interest before 2006.

In the subsequent sections, we provide an overview of the DQ definitions, DQ dimensions, their interrelationships, and DQ outcomes to develop a consolidated digital health DQ framework.

### DQ Definitions

Multiple definitions of DQ were discussed in the literature (Multimedia Appendix 5 [17,18,20-22,31,54,67-77]). There was no consensus on a single definition of DQ; however, an analysis of the definitions revealed two perspectives, which we labeled as the (1) context-agnostic perspective and (2) context-aware perspective. The context-agnostic perspective defines DQ based on a set of dimensions, regardless of the context within which the data are used. For instance, as Abiy et al [67] noted “documentation and contents of data within an electronic medical record (EMR) must be accurate, complete, concise, consistent and universally understood by users of the data, and must support the legal business record of the organization by maintaining the required parameters such as consistency, completeness and accuracy.” By contrast, the context-aware perspective evaluates the dimensions of DQ with recognition of the context within which the data are used. For instance, as the International Organization for Standardization and Liu et al [78] noted, DQ is “the degree to which data satisfy the requirements defined by the product-owner organization” and can be reflected through its dimensions such as completeness and accuracy.

### DQ Dimensions

#### Overview

In total, 30 subthemes were identified and grouped into 6 DQ dimensions: accuracy, consistency, completeness, contextual validity, accessibility, and currency (Table 1; Multimedia Appendix 6 [8-12,14-16,18-22,31-62,67,69,71,72,76,79-168]). Consistency (164/227, 72.2%), completeness (137/227, 60.4%), and accuracy (123/227, 54.2%) were the main DQ dimensions. Comparatively, less attention was paid to accessibility (28/227, 12.3%), currency (18/227, 7.9%), and contextual validity (26/227. 11.5%).

**Table 1:** Description of the data quality (DQ) dimensions.

Dimension	Description	Subthemes
Accuracy	“The degree to which data reveal the truth about the event being described” [31]	Validity, correctness, integrity, conformance, plausibility, veracity, and accurate diagnostic data
Consistency	“Absence of differences between data items representing the same objects based on specific information requirements. Consistent data contain the same data values when compared between different databases” [31]	Inconsistent data capturing, standardization, concordance, uniqueness, data variability, temporal variability, system differences, semantic consistency, structuredness, and representational consistency
Completeness	“The absence of data at a single moment over time or when measured at multiple moments over time” [79]	Missing data, level of completeness, representativeness, fragmentation, and breadth of documentation
Contextual validity	Assessment of DQ is “dependent on the task at hand” [18]	Contextual DQ, fitness for use, granularity, and relevancy
Accessibility	How “feasible it is for users to extract the data of interest” [18]	Accessible DQ and availability
Currency	“The degree to which data represent reality from the required point in time” [32]	Timeliness

#### DQ Dimension: Accessibility

The accessibility dimension (28/227, 12.3%) is composed of both the accessibility (15/28, 54%) and availability (13/28, 46%) subthemes, reflecting the feasibility for users to extract data of interest [18]. Scholars regularly view the *accessibility* subtheme favorably, with the increased adoption of electronic health record (EHR) systems overcoming physical and chronological boundaries associated with paper records by allowing access to information from multiple locations at any time [33,80]. Top et al [33] noted that EHR made it possible for nurses to access patient data, resulting in improved decision-making. Furthermore, Rosenlund et al [81] noted that EHRs benefit health care professionals by providing increased opportunities for searching and using information. The *availability* subtheme is an extension of the accessibility subtheme and examines whether data exist and whether the existing data are in a format that is readily usable [34]. For instance, Dentler et al [34] noted that pathology reports, although accessible, are recorded in a nonstructured, free-text format, making it challenging to readily use the data. Although structuredness may make data more available, Yoo et al [82] highlighted that structured data entry in the form of drop-down lists and check boxes tends to reduce the narrative description of patients’ medical conditions. Although not explicitly investigating accessibility, Makeleni and Cilliers [31] also noted the challenges associated with structured data entry.

#### DQ Dimension: Accuracy

The accuracy dimension (123/227, 54.2%) is composed of 7 subthemes, namely correctness (42/123, 34.1%), validity (23/123, 18.7%), integrity (19/123, 15.4%), plausibility (17/123, 13.8%), accurate diagnostic data (13/123, 10.6%), conformance (7/123, 5.7%), and veracity (2/123, 1.6%). Accuracy refers to the extent to which data reveal the truth about the event being described [31] and conform to their actual value [83].

Studies often referred to accuracy as the “*correctness”* of data, which is the degree to which data correctly communicate the parameter being represented [32]. By contrast, other studies focused on *plausibility*, which is the extent to which data points are believable [35]. Although accuracy concerns were present for all forms of digital health data, some studies focused specifically on *inaccuracies in diagnostic data* and stated that “the accurate and precise assignment of structured [diagnostic] data within EHRs is crucial” [84] and is “key to supporting secondary clinical data” [36].

To assess accuracy, the literature regularly asserts that data must be *validated* against metadata constraints, system assumptions, and local knowledge [19] and *conform* to structural and syntactical rules. According to Kahn et al [19] and Sirgo et al [85], conformance focuses on the compliance of data with internal or external formatting and relational or computational definitions. Accurate, verified, and validated data as well as data conforming to standards contribute to the *integrity* of the data. Integrity requires that the data stored in health information systems are accurate and consistent, where the “improper use of [health information systems] can jeopardise the integrity of a patient’s information” [31]. An emerging subtheme of accuracy is the veracity of data, which represents uncertainty in the data owing to inconsistency, ambiguity, latency, deception, and model approximations [21]. It is particularly important in the context of the secondary use of big data, where “data veracity issues can arise from attempts to preserve privacy,...and is a function of how many sources contributed to the data” [86].

#### DQ Dimension: Completeness

The completeness dimension (114/227, 50.2%) is composed of 5 subthemes: missing data (66/114, 57.9%), level of completeness (25/114, 21.9%), representativeness (13/114, 11.4%), fragmentation (8/114, 7%), and breadth of documentation (2/114, 1.8%). A well-accepted definition of data completeness considers 4 perspectives: documentation (the presence of observations regarding a patient in data), breadth (the presence of all desired forms of data), density (the presence of a desired frequency of data values over time), and prediction (the presence of sufficient data to predict an outcome) [169]. Our analysis revealed that these 4 perspectives, although accepted, are rarely systematically examined in the extant literature; rather, papers tended to discuss completeness or the lack thereof as a whole.

*Missing* data is a prominent subtheme and represents a common problem in EHR data. For instance, Gloyd et al [87] argued that incomplete, missing, and implausible data “was by far the most common challenge encountered.” Scholars regularly identified that data fragmentation contributed to incompleteness, with a patient’s medical record deemed incomplete owing to data being required from multiple systems and EHRs [18,37,88-93]. “Data were also considered hidden within portals, outside systems, or multiple EHRs, frustrating efforts to assemble a complete clinical picture of the patient” [89]. Positive perspectives pertaining to data completeness focus on the level of completeness, with studies reporting relatively high completeness rates in health data sets [34,38,80,94,95,170]. For data to be considered complete, it needs to be captured at sufficient breadth and depth over time [12,18].

Some studies have proposed techniques for improving completeness, including developing fit-for-purpose user interfaces [68,96,97], standardizing documentation practices, [98,99], automating documentation [100], and performing quality control [99].

In some instances, the *level of completeness* and *extent of missing data* differed depending on the health status of the patient [15,16,18,20,39-43,86,90,101,170,171], which we classified into the subtheme of *representativeness*. It has been found that there is “a statistically significant relationship between EHR completeness and patient health status” [42], with more data recorded for patients who are sick than for patients with less-acute conditions. This strongly aligns with the subtheme of contextual validity.

#### DQ Dimension: Consistency

The consistency dimension (157/227, 69.2%) is composed of 10 subthemes: inconsistent data capturing (33/157, 21%), standardization (28/157, 17.8%), concordance (22/157, 14%), uniqueness (14/157, 8.9%), data variability (14/157, 8.9%), temporal variability (13/157, 8.3%), system differences (12/157, 7.6%), semantic consistency (10/157, 6.4%), structuredness (7/157, 4.5%), and representational consistency (4/157, 2.5%).

*Inconsistent data capturing* is a prevalent subtheme caused by the manual nature of data entry in health care settings [86], especially when data entry involve multiple times, teams, and goals [102]. Inconsistent data capturing results in *data variability* and *temporal variability*. Data variability refers to inconsistencies in the data captured within and between health information systems, whereas temporal variability reflects inconsistencies that occur over time and may be because of changes in policies or medical guidelines [20,44-46,87,103-105]. *Semantic inconsistency* (ie, data with logical contradictions) and *representational inconsistency* (ie, data variations owing to multiple formats) can also result from inconsistent data capturing [47].

*Standardization in terms of* terminology, diagnostic codes, and workflows [99] are proffered to minimize inconsistency in data entry, yet in practice, there is a “lack of standardized data and terminology” [9] and “even with a set standard in place not all staff accept and follow the routine” [99]. The lack of standardization is further manifested because of health information *system differences* across settings [106]. As a result of the differences between systems, *concordance*—the extent of “agreement between elements in the EHR, or between the EHR and another data source”—is hampered [107].

Furthermore, inconsistent data entry can be caused by redundancy within the system because of structured versus unstructured data [108], which we label as the subtheme “*structuredness*,” and duplication across systems [39,48,104,109,172,173], which we label as the subtheme “*uniqueness*.” Although structured data entry “facilitates information retrieval” [33] and is “in a format that enables reliable extraction” [18], the presence of unstructured fields leads to data duplication efforts, hampering uniqueness, as data are recorded in multiple places with varying degrees of granularity and level of detail.

#### DQ Dimension: Contextual Validity

The contextual validity dimension (26/227, 11.5%) is composed of 4 subthemes: fitness for use (11/26, 42%), contextual DQ (9/26, 35%), granularity (4/26, 15%), and relevancy (2/26, 8%). Contextual validity requires a deep understanding of the context that gives rise to data [86], including technical, organizational, behavioral, and environmental factors [174].

*Contextual DQ* is often described as “fitness of use” [20], for which understanding the context in which data are collected is deemed important [18,90]. Another factor that contributes to data being fit for use is the *granularity* of data. Adequate *granularity* of time stamps [49], patient information [16], and data present in EHR (eg, diagnostic code [16]) was considered important to make data fit for use. Finally, for data to be fit for use, they must be *relevant*. As indicated by Schneeweiss and Glynn [41], for data to be meaningful, health care databases need to contain relevant information of sufficient quality, which can help answer specific questions. The literature clearly demonstrates the need to take context into consideration when analyzing data and the need to adapt technologies to the health care context so that appropriate data are collected for reliable analysis to be performed.

#### DQ Dimension: Currency

The currency dimension (18/227, 7.9%) is composed of a single subtheme: *timeliness*. Currency, or timeliness, is defined by Afshar et al [32] and Makeleni and Cilliers [31] as the degree to which data represent reality from the required point in time. From an EHR perspective, data should be up to date, available, and reflect the profile of the patient at the time when the data are accessed [32,50]. Lee et al [35] extended this to include the recording of an event at the time when it occurs such that a value is deemed current if it is representative of the clinically relevant time of the event. Frequently mentioned causes for lack of currency of data include (1) recording of events (long) after the event actually occurred [91,99,110,111], (2) incomplete recording of patient characteristics over time [16], (3) system or interface design not matching workflow and impeding timely recording of data [99], (4) mixed-mode recording—paper and electronic [99], and (5) lack of time stamp metadata, meaning that the temporal sequence of events is not reflected in the recorded data [16].

#### Interrelationships Among the DQ Dimensions

**Figure 4:**
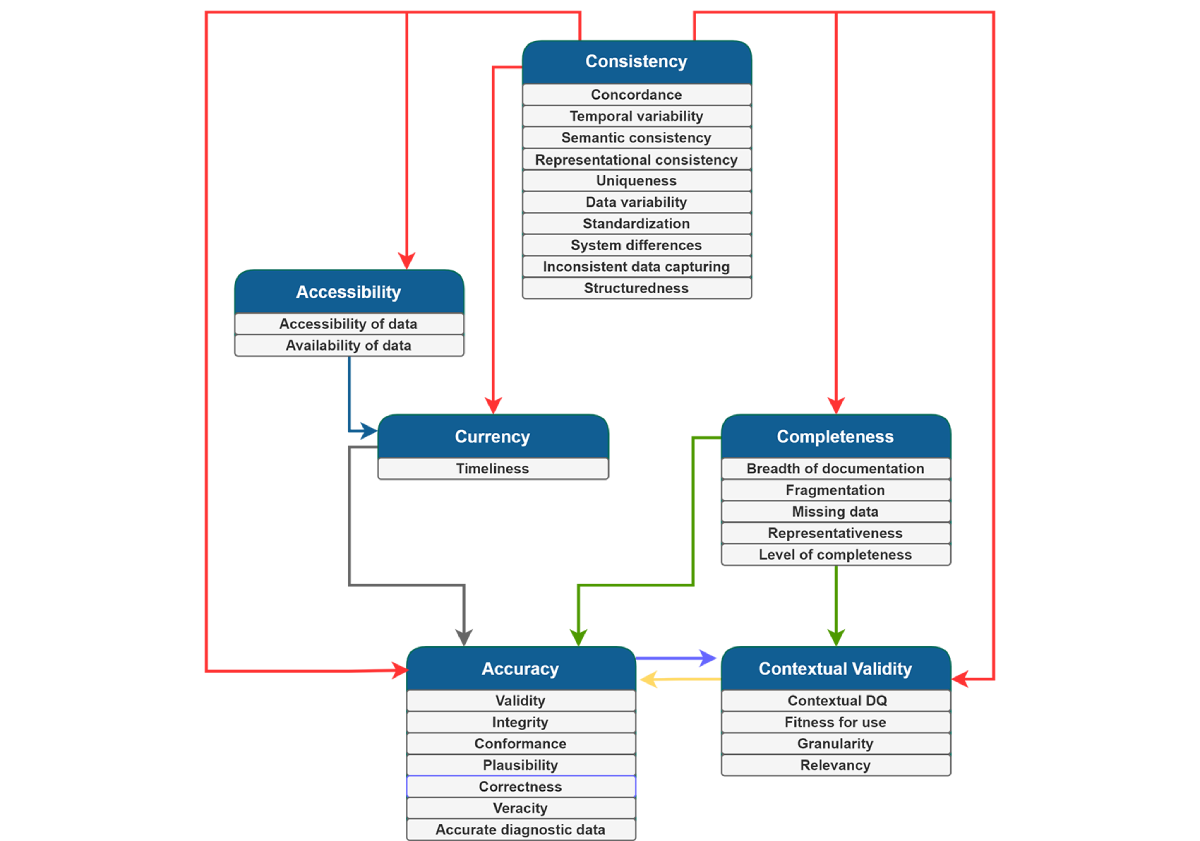
Interrelationships between the data quality (DQ) dimensions.

As illustrated in Figure 4 and Multimedia Appendix 7 [16,34,40,42,78,80,90,91,109], interrelationships were found among the digital health DQ dimensions.

Consistency influenced all the DQ dimensions. Commonly, these relationships were expressed in terms of the presence of structured and consistent data entry, which prompts complete and accurate data to be entered into the health information system and provides more readily accessible and current data for health care professionals when treating patients. As Roukema et al [80] noted, “structured data entry applications can prompt for completeness, provide greater accuracy and better ordering for searching and retrieval, and permit validity checks for DQ monitoring, research, and especially decision support.” When data are entered inconsistently, it impedes the accuracy of the medical record and the contextual validity for secondary uses of the data [40].

Accessibility of data was found to influence the currency dimension of DQ. When data are not readily accessible, they seldom satisfy the timeliness of information for health care or research purposes [34]. Currency also influenced the accuracy of data. In a study investigating where DQ issues in EHR arise, it was found that “false negatives and false positives in the problem list sometimes arose when the problem list...[was] out-of-date, either because a resolved problem was not removed or because an active problem was not added” [90].

Furthermore, completeness influences the accuracy of data; as Makeleni and Cilliers [31] noted, “data should be complete to ensure it is accurate.” The presence of inaccurate data was regularly linked to information fragmentation [88], incomplete data entry [109], and omissions [35]. Completeness also influenced contextual validity, as it is necessary to have all the data available to complete specific tasks [78]. When it comes to the secondary use of EHR data, evaluation of “completeness becomes extrinsic, and is dependent upon whether or not there are sufficient types and quantities of data to perform a research task of interest” [42].

Accuracy and contextual validity exhibited a bidirectional relationship with each other. The literature suggests that accuracy influences contextual validity; however, data cannot simply be extracted from structured form fields, and free-text fields will also need to be consulted. For instance, Kim and Kim [112] identified “it is sometimes thought that structured data are more completely optimized for clinical research. However, this is not always the case, particularly given that extracted EMR data can still be unstable and contain serious errors.” By contrast, other studies suggest that when only a segment of information regarding a specific clinical event (ie, contextual validity) is captured, inaccuracy can ensue [16].

### Outcomes of Digital Health DQ

**Figure 5:**
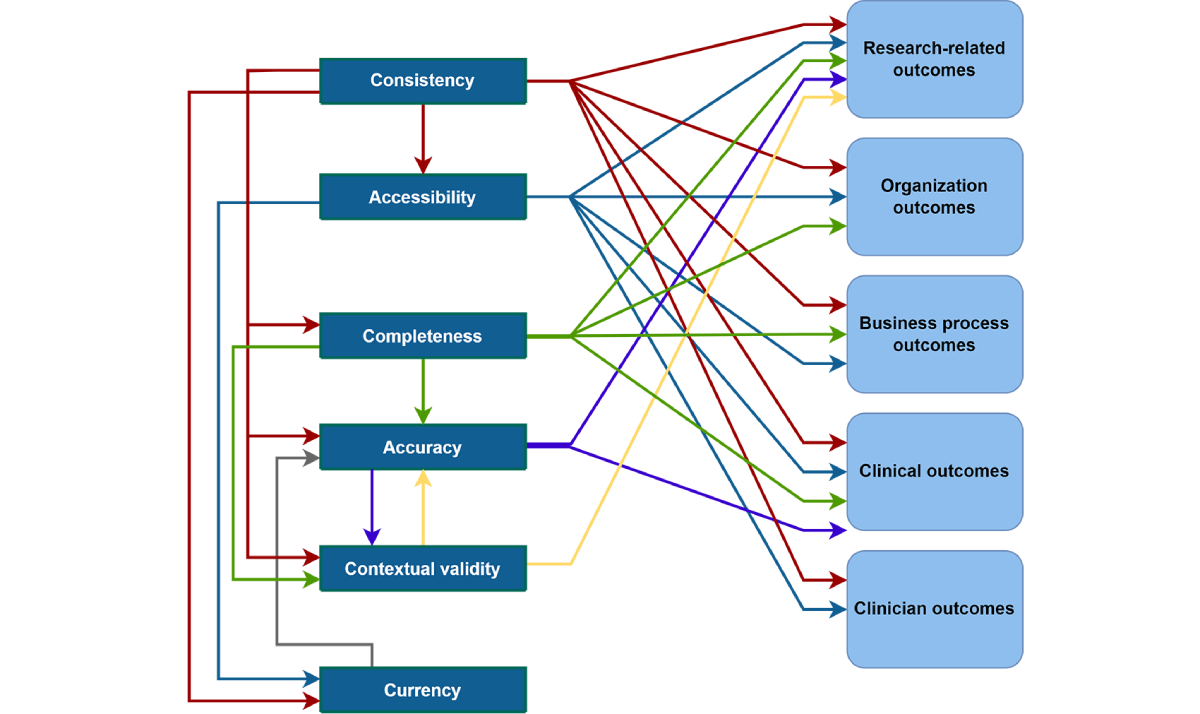
Consolidated digital health data quality dimension and outcome framework.

The analysis of the literature identified five types of digital health DQ outcomes: (1) clinical, (2) business process, (3) clinician, (4) research-related, and (5) organizational outcomes (Multimedia Appendix 8 [15,16,20,31,33,39,40,42,51,52,55,57, 58,61,63,64,84,90,105,113,166,175-178]). Using NVivo’s built-in cross-tab query coupled with subject matter expert analysis, it was identified that different DQ dimensions were related to DQ outcomes in different ways (Table 2). Currency was the only dimension that did not have a direct effect on DQ outcomes. However, as shown in Figure 5, it is plausible that currency affects DQ outcomes by impacting other DQ dimensions. In the subsequent paragraphs, we discuss each DQ dimension and its respective outcomes.

We identified that the accessibility DQ dimension influenced clinical, clinician, business process, research-related, and organizational outcomes. In terms of *clinical outcomes,* Roukema et al [80] indicated that EHRs substantially enhance the quality of patient care by improving the accessibility and legibility of health care data. The increased accessibility of medical records during the delivery of patient care is further proffered to benefit *clinicians* by reducing the data entry burden [33]. By contrast, inconsistency in the availability of data across health settings increases clinician workload; as Wiebe et al [15] noted, “given the predominantly electronic form of communication between hospitals and general practitioners in Alberta, the inconsistency in availability of documentation in one single location can delay processes for practitioners searching for important health information.” When data are accessible and available, they can improve *business processes* (eg, quality assurance) and *research*-*related* (eg, outcome-oriented research) *outcomes* and can support *organizational outcomes* with improved billing and financial management [179].

The literature demonstrates that data accuracy influences *clinical outcomes* [14,39,51] and *research-related outcomes* [14,113]; as Wang et al [14] described, “errors in healthcare data are numerous and impact secondary data use and potentially patient care and safety.” Downey et al [39] observed the negative impact on quality of care (ie, *clinical outcomes)* resulting from incorrect data and stated, “manual data entry remains a primary mechanism for acquiring data in EHRs, and if the data is incorrect then the impact to patients and patient care could be significant” [39]. Poor data accuracy also diminishes the quality of *research outcomes*. Precise data are beneficial in producing high-quality research outcomes. As Gibby [113] explained, “computerized clinical information systems have considerable advantages over paper recording of data, which should increase the likelihood of their use in outcomes research. Manual records are often inaccurate, biased, incomplete, and illegible.” Closely related to accuracy, contextual validity is an important DQ dimension that considers the fitness for *research*; as stated by Weiskopf et al [42], “[w]hen repurposed for secondary use, however, the concept of ‘fitness for use’ can be applied.”

The consistency DQ dimension was related to all DQ outcomes. It was commonly reported that inconsistency in data negatively impacts the *reusability* of EHR data for research purposes, hindering *research-related outcomes* and negatively impacting *business processes* and *organizational outcomes*. For example, Kim et al [114] acknowledged that inconsistent data labeling in EHR systems may hinder accurate research results, noting that “a system may use local terminology that allows unmanaged synonyms and abbreviations...If local data are not mapped to terminologies, performing multicentre research would require extensive labour.” Alternatively, von Lucadou et al [16] indicated the impact of inconsistency on *clinical outcomes*, reporting that the existence of inconsistencies in captured data “could explain the varying number of diagnoses throughout the encounter history of some subjects,” whereas Diaz-Garelli et al [84] demonstrated the negative impact that inconsistency has on *clinicians* in terms of increased workload.

Incomplete EMR data were found to impact *clinical outcomes* (eg, reduced quality of care), *business process outcomes* (eg, interprofessional communication), *research-related outcomes* (eg, research facilitation), and *organizational outcomes* (eg, key performance indicators related to readmissions) [15]. For example, while reviewing the charts of 3011 nonobstetric inpatients, Wiebe et al [15] found that missing discharge summary within an EHR “can present several issues for healthcare processes, including hindered communication between hospitals and general practitioners, heightened risk of readmissions, and poor usability of coded health data,” among other widespread implications. Furthermore, Liu et al [69] reported that “having incomplete data on patients’ records has posed the greatest threat to patient care.” Owing to the heterogeneous nature (with multiple data points) of EHR data, Richesson et al [20] emphasized that access to large, complete data will allow clinical investigators “to detect smaller clinical effects, identify and study rare disorders, and produce robust, generalisable results.”

**Table 2:** The relationships between *data quality (DQ)* dimensions and data outcomes.

DQ dimension	Outcomes^a^				
	Research	Organizational	Business process	Clinical	Clinician
Accessibility	✓	✓	✓	✓	✓
Accuracy	✓			✓	
Completeness	✓	✓	✓	✓	
Consistency	✓	✓	✓	✓	✓
Contextual validity	✓				
Currency					

^a^The checkmark symbol indicates that the relationship between the DQ dimension and the outcome is reported in the literature. Blank cells indicate that there is no evidence to support the relationship.

## Discussion

### Overview

The following sections describe the three main findings of this research: the (1) dimensions of DQ, (2) interrelationships among the dimensions of DQ, and (3) outcomes of DQ. As described in the *Summary of Key Findings* section, these 3 findings led to the development of the DQ-DO framework. Subsequently, we compared the DQ-DO framework with related works. This leads to implications for future research. The *Discussion* section concludes with a reflection on the limitations of this study.

### Summary of Key Findings

In summary, we unearthed 3 core findings. First, we identified 6 dimensions of DQ within the digital health domain: consistency, accessibility, completeness, accuracy, contextual validity, and currency. These dimensions were synthesized from 30 subthemes described in the literature. We found that consistency, completeness, and accuracy are the predominant dimensions of DQ. Comparatively, limited attention has been paid to the dimensions of accessibility, currency, and contextual validity. Second, we identified the interrelationships among these 6 dimensions of digital health DQ (Table 2). The literature indicates that the consistency dimension can influence all other DQ dimensions. The accessibility of data was found to influence the currency of data. Completeness impacts accuracy and contextual validity, with these dimensions serving as dependent variables and exhibiting a bidirectional relationship with each other. Third, we identified 5 types of data outcomes (Table 2; Multimedia Appendix 8): research-related, organizational, business process, clinical, and clinician outcomes. Consistency was found to be a very influential dimension, impacting all types of DQ outcomes. By contrast, contextual validity was shown to be particularly important for data reuse (eg, performance measurement and outcome-oriented research). Although currency does not directly impact any outcomes, it impacts the accuracy of data, which impacts clinical and research-related outcomes. Therefore, if currency issues are not resolved, accuracy issues would still prevail. Consistency, accessibility, and completeness were shown to be important considerations for achieving the goal of improving organizational outcomes. Through consolidating our 3 core findings, we developed a consolidated DQ-DO framework (Figure 5).

### Comparison With Literature

Our findings extend those of previous studies on digital health DQ in 3 ways. First, through our rigorous approach, we identified a comprehensive set of DQ dimensions, which both confirmed and extended the existing literature. For instance, Weiskopf and Weng [17] identified 5 DQ dimensions, namely completeness, correctness, concordance, plausibility, and currency, all of which are present within our DQ framework, although in some instances, we use slightly different terms (referring to correctness as accuracy and concordance as consistency). Extending the framework of Weiskopf and Weng [17], we view plausibility as a subtheme of accuracy and disentangle accessibility from completeness, and we also stress the importance of contextual validity per Richesson et al [20]. Others have commonly had a narrower perspective of DQ, focusing on completeness, correctness, and currency [18] or on completeness, timeliness, and accuracy [13]. In other domains of digital health, such as physician rating systems, Wang and Strong’s [180] DQ dimensions of intrinsic, contextual, representational, and accessibility have been adopted. Such approaches to assessing DQ are appropriate, although they remove a level of granularity that is necessary to understand relationships and outcomes. This is particularly necessary given the salience of consistency in our data set and the important role it plays in generating outcomes.

Second, unlike previous studies on DQ dimensions, we also demonstrate how these dimensions are all related to each other. By analyzing the interrelationships between these DQ dimensions, we can determine how a particular dimension influences another and in which direction this relationship is unfolding. This is an important implication for digital health practitioners, as although several studies have examined how to validate [38] and resolve DQ issues [16], resolving issues with a specific DQ dimension requires awareness of the interrelated DQ dimensions. For instance, to improve accuracy, one also needs to consider improving consistency and completeness.

Third, although previous studies describe how DQ can impact a particular outcome (eg, the studies by Weiskopf et al [18], Johnson et al [52], and Dantanarayana and Sahama [115]), they largely focus broadly on DQ, a specific dimension of DQ, or a specific outcome. For instance, Sung et al [181] noted that poor-quality data were a prominent barrier hindering the adoption of digital health systems. By contrast, Kohane et al [182] focused on research-related outcomes in terms of publication potential and identified that incompleteness and inconsistency can serve as core impediments. To summarize, the DQ-DO framework (Figure 5) developed through this review provides not only the dimensions and the outcomes but also the interrelationships between these dimensions and how they influence outcomes.

### Implications for Future Work

#### Implication 1: Equal Consideration Across DQ Dimensions

This study highlights the importance of each of the 6 DQ dimensions: consistency, accessibility, completeness, accuracy, contextual validity, and currency. These dimensions have received varying amounts of attention in the literature. Although we observe that some DQ dimensions such as accessibility, contextual validity, and currency are discussed less frequently than others, it does not mean that these dimensions are not important for assessment. This is evident in Figure 5, which shows that all DQ dimensions except for currency directly influence DQ outcomes. Although we did not identify a direct relationship between the currency of data and the 6 types of data outcomes, it is likely that the currency of data influences the accuracy of data, which subsequently influences the research-related and clinical outcomes. Future research, including consultation with a range of stakeholders, needs to further delve into understanding the underresearched DQ dimensions. For instance, both the currency and accessibility of data are less frequently discussed dimensions in the literature; however, with the advances in digital health technologies, both have become highly relevant for real-time clinical decisions [21,53].

#### Implication 2: Empirical Investigations of the Impact of the DQ Dimensions

The DQ-DO framework identified in this study has been developed through a rigorous systematic literature review process, which synthesized the literature related to digital health DQ. To extend this study, we advocate for empirical mixed methods case studies to validate the framework, including an examination of the interrelationships between DQ dimensions and DQ outcomes, based on real-life data and consultation with a variety of stakeholders. Existing approaches can be used to identify the presence of issues related to DQ dimensions within digital health system logs [38,183]. The DQ outcomes could be assessed by extracting prerecorded key performance indicators from case hospitals and be triangulated with interview data to capture patients’, clinicians’, and hospitals’ perspectives of the impacts of DQ [184]. This could then be incorporated into a longitudinal study in which data collection is performed before and after a DQ improvement intervention, which would provide efficacy to the digital health DQ intervention.

#### Implication 3: Understanding the Root Causes of DQ Challenges

Although this study provides a first step toward a more comprehensive understanding of DQ dimensions for digital health data and their influences on outcomes, it does not explore the potential causes of such DQ challenges. Without understanding the reasons behind these DQ issues, the true potential of evidence-based health care decision-making remains unfulfilled. Future research should examine the root causes of DQ challenges in health care data to prevent such challenges from occurring in the first place. A framework that may prove useful in illuminating the root causes of DQ issues is the Odigos framework, which indicates that DQ issues emanate from the social world (ie, macro and situational structures, roles, and norms), material world (eg, quality of the EHR system and technological infrastructure), and personal world (eg, characteristics and behaviors of health care professionals) [183]. These insights could then be incorporated into a data governance roadmap for digital hospitals.

#### Implication 4: Systematic Assessment and Remedy of DQ Issues

Although prevention remains better than the cure (refer to implication 3), not all DQ errors can be prevented or mitigated. It is common for many health care organizations to dedicate resources to data cleaning to obtain high-quality data in a timely manner, and this will remain necessary (although hopefully to a lesser degree). Some studies (eg, Weiskopf et al [18]) advocate evidence-based guidelines and frameworks for a detailed assessment of the quality of digital health data. However, few studies have focused on a systematic and automated method of assessing and remedying common DQ issues. Future research should also focus on evidence-based guidelines, best practices, and automated means to assess and remedy digital health data.

### Limitations

This review is scoped to studying digital health data generated within a hospital setting and not those generated within other health care settings. This is necessary because of the vast differences between acute health care settings and primary care. Future research should seek to investigate the digital health data of primary care settings to identify the DQ dimensions and outcomes relevant to these settings. In addition, this literature review has been scoped to peer-reviewed outlets, with “grey” literature excluded, which could have led to publication bias. Although this scoping may have resulted in the exclusion of some relevant articles, it was necessary to ensure the quality behind the development of the digital health DQ framework. An additional limitation that may be raised by our method is that because of the sheer number of articles returned by our search, we did not perform double coding (where 2 independent researchers analyze the same article). To mitigate this limitation, steps were taken to minimize bias by conducting coder corroboration sessions and group validation, as mentioned in the *Methods* section, with the objective of improving internal and external reliability [66]. To further improve internal reliability, 2 experienced researchers verified the entirety of the analysis in NVivo and to improve external reliability, card-sorting assessments were performed with DQ experts, and the findings were presented and confirmed by 3 digital health care professionals. Furthermore, empirical validation of the framework is required, both in terms of real-life data and inputs from a range of experts.

### Conclusions

The multidisciplinary systematic literature review conducted in this study resulted in the development of a consolidated digital health DQ framework comprising 6 DQ dimensions, the interrelationships among these dimensions, 6 DQ outcomes, and the relationships between these dimensions and outcomes. We identified four core implications to motivate future research: specifically, researchers should (1) pay equal consideration to all dimensions of DQ, as the dimensions can both directly and indirectly influence DQ outcomes; (2) seek to empirically assess the DQ-DO framework using a mixed methods case study design; (3) identify the root causes of the digital health DQ issues; and (4) develop interventions for mitigating DQ issues or preventing them from arising. The DQ-DO framework provides health care executives (eg, chief information officers and chief clinical informatics officers) with insights into DQ issues and which digital health-related outcomes they have an impact on, and this can help them prioritize tackling DQ-related problems.

## Appendix

Multimedia Appendix 1 Description of key terms.

Multimedia Appendix 2 Verification of search strategy.

Multimedia Appendix 3 Data coding structures.

Multimedia Appendix 4 Publication outlets.

Multimedia Appendix 5 Data quality definitions.

Multimedia Appendix 6 Evidence of the subtheme for each data quality dimension.

Multimedia Appendix 7 Evidence for the interrelationships among the dimensions of data quality.

Multimedia Appendix 8 Evidence for the outcomes of data quality.

## Abbreviations

DQ: data quality
DQ-DO: data quality dimension and outcome
EHR: electronic health record
EMR: electronic medical record
PRISMA: Preferred Reporting Items for Systematic Reviews and Meta-Analyses
