# Supplementary material for: Digital Health Data Quality Issues: Systematic Review
Source: J Med Internet Res. 2023 Mar 31;25:e42615. doi: 10.2196/42615 (PMC10131725; doi:10.2196/42615)
Supplement: Multimedia Appendix 1 [file jmir_v25i1e42615_app1.docx]

**Table S1.** Description of common acronyms used in the study.

| Acronym | Explication | Description |
| --- | --- | --- |
| DQ | Data quality | The extent to which digital health data are accessible, accurate, complete, consistent, contextually valid, and current. |
| DQ dimensions | Data quality dimensions | The components used to evaluate data quality (ie, accessibility, accuracy, completeness, consistency, contextual validity, and currency) |
| DQ-DO framework | Data quality-data outcomes framework | The consolidated framework developed in this study demonstrating the interrelationships between data quality dimensions and their relationships with data quality outcomes. |
| EHR | Electronic health record | A longitudinal and electronic collection of patients’ clinical information available across case settings [24]. |
| EMR | Electronic medical record | Synonymous to EHR. |
