# Supplementary material for: Digital Health Data Quality Issues: Systematic Review
Source: J Med Internet Res. 2023 Mar 31;25:e42615. doi: 10.2196/42615 (PMC10131725; doi:10.2196/42615)
Supplement: Multimedia Appendix 2 [file jmir_v25i1e42615_app2.docx]

## Appendix 2: Verification of Search Strategy

| Area | Researchers (CIs) | | Subject Expert 1 | | Reference Librarian | | Subject Expert 2 | | Co-Researchers | |
| --- | --- | --- | --- | --- | --- | --- | --- | --- | --- | --- |
| Research Questions |  | R |  | C,V |  | . |  | V |  | C |
| Keywords |  | R |  | V |  | C |  | V |  | C |
| Subject Area /Domain |  | R |  | C, V |  | . |  | V |  | C |
| Search Databases |  | R |  | V |  | V |  | V |  | C |
| Journals |  | R |  | C, V |  | V |  | V |  | C |
| Conferences |  | R |  | C, V |  | V |  | V |  | C |
| Search Engine |  | R |  | . |  | V |  | . |  | R |
| Relevance of Selected Seminal Articles |  | R |  | V |  | . |  | V |  | V |
| R= Responsible, V= Verifier, C = Contributor | | | | | | | | | | |
