# Supplementary figures and images for: Digital Health Data Quality Issues: Systematic Review
Source: J Med Internet Res. 2023 Mar 31;25:e42615. doi: 10.2196/42615 (PMC10131725; doi:10.2196/42615)

Appendix 3: Data Coding Structures


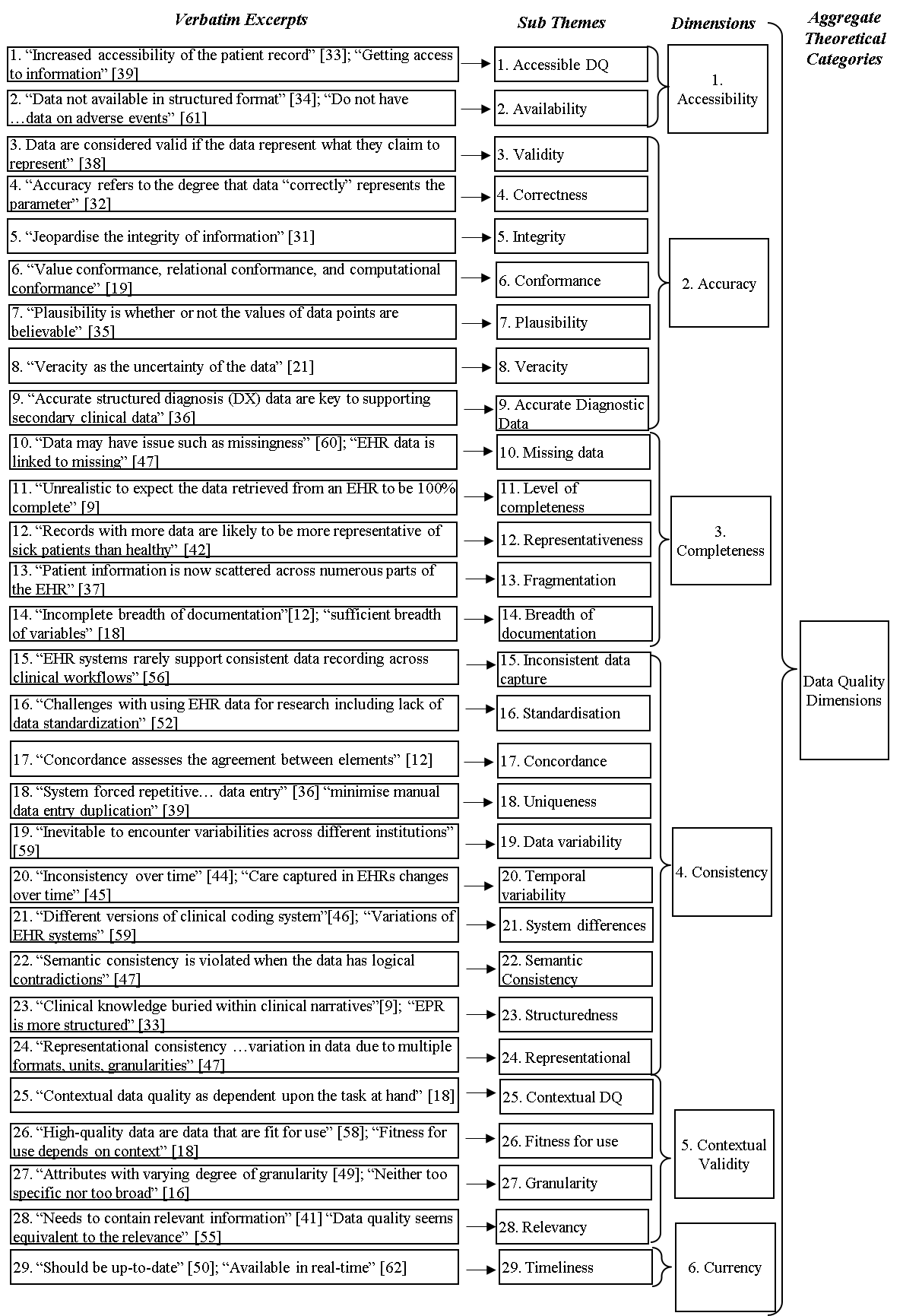


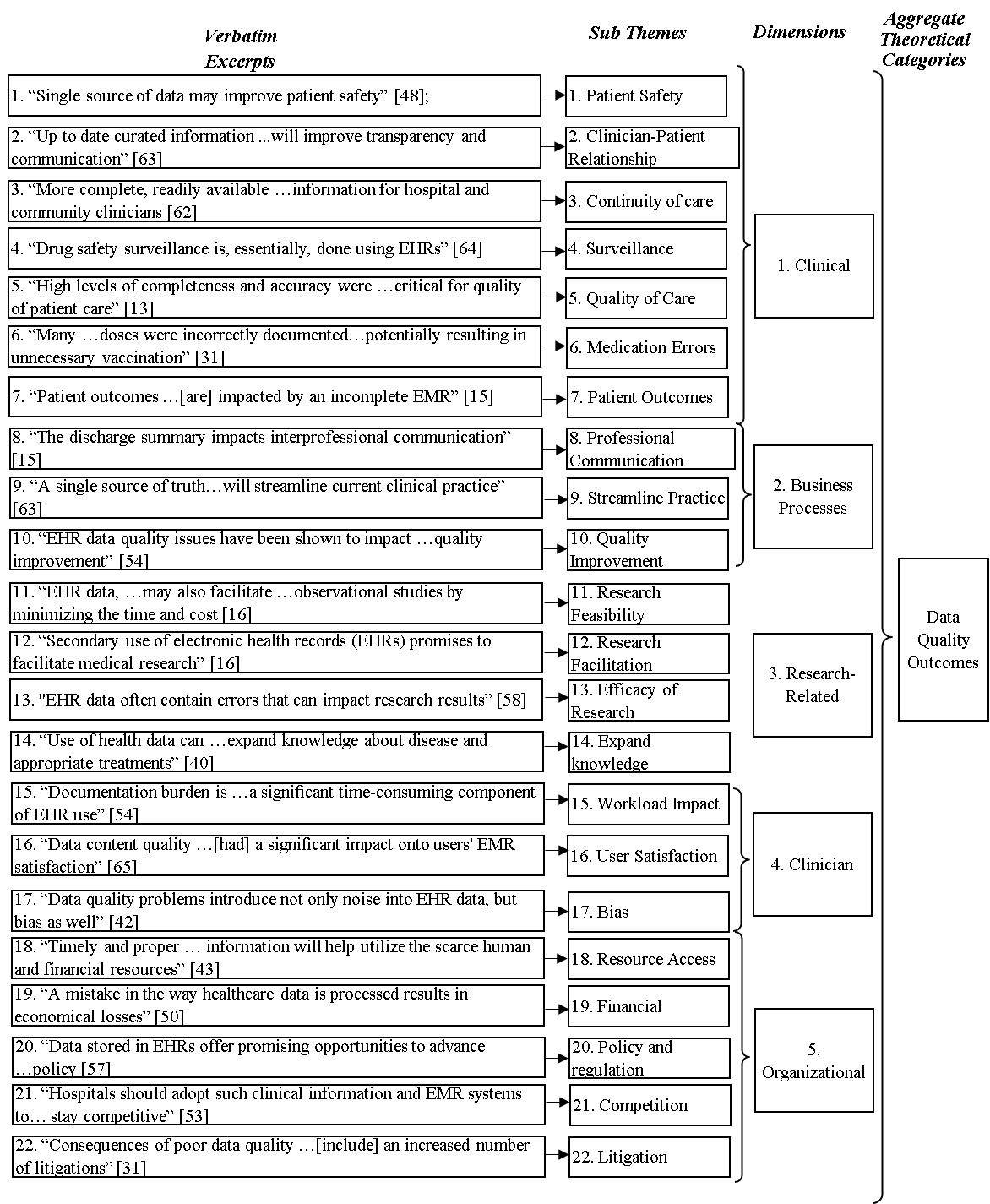

Supplement: Multimedia Appendix 3 [file jmir_v25i1e42615_app3.docx]
