# Supplementary material for: Digital Health Data Quality Issues: Systematic Review
Source: J Med Internet Res. 2023 Mar 31;25:e42615. doi: 10.2196/42615 (PMC10131725; doi:10.2196/42615)
Supplement: Multimedia Appendix 4 [file jmir_v25i1e42615_app4.docx]

## Appendix 4: Publication outlets

| **Outlet** | **N*** |
| --- | --- |
| Abdominal Radiology | 1 |
| American Journal of Emergency Medicine | 1 |
| American Journal of Law and Medicine | 1 |
| America's Conference on Information Systems 2017 | 1 |
| AMIA Annual Symposium | 16 |
| AMIA Joint Summits on Translational Science Proceedings | 2 |
| Anesthesia and analgesia | 1 |
| Anesthesiology Clinics | 1 |
| Annals of Internal Medicine | 1 |
| Applied Clinical Informatics | 4 |
| Applied Network Science | 1 |
| Asian Bioethics Review | 1 |
| Asia-Pacific Conference on Business Process Management | 1 |
| Australasian Computer Science Week 2016 | 1 |
| Australasian Conference on Information Systems | 2 |
| Australian Health Review | 2 |
| BioMedicine | 1 |
| BMC Emergency Medicine | 3 |
| BMC Health Services Research | 2 |
| BMC Infectious Diseases | 1 |
| BMC Medical Informatics and Decision Making | 11 |
| BMC Medical Research Methodology | 1 |
| BMC Medicine | 1 |
| BMC Pediatrics | 1 |
| BMJ | 1 |
| BMJ Open | 2 |
| Building Capacity for Health Informatics in the Future | 1 |
| Building Continents of Knowledge in Oceans of Data: The Future of Co-Created eHealth | 1 |
| Business & Information Systems Engineering | 1 |
| Canadian Journal of Diabetes | 1 |
| Clinical Epidemiology | 1 |
| Computer Methods and Programs in Biomedicine | 3 |
| Computers in Biology and Medicine | 1 |
| Decision Support Systems | 2 |
| Deeble Institute for Health Policy Research | 1 |
| Digital Personalized Health and Medicine | 2 |
| eGEMs | 8 |
| e-Health – For Continuity of Care | 1 |
| Electronic Journal of Health Informatics | 1 |
| Emergency Medicine Australasia | 1 |
| Endocrinol Metabolism | 1 |
| European Journal of Cardio-thoracic Surgery | 1 |
| Frontiers in Medicine | 1 |
| German Medical Data Sciences: Bringing Data to Life | 1 |
| GigaScience | 1 |
| Government Information Quarterly | 1 |
| Hawaii International Conference on System Sciences | 4 |
| Health Informatics Journal | 2 |
| Health Information Management Journal | 1 |
| Health Policy and Technology | 1 |
| Health Research Policy and Systems | 1 |
| Health Services Research | 1 |
| Healthcare | 1 |
| Healthcare Executive | 1 |
| Healthcare Quarterly | 1 |
| Healthcare Technology Letters | 1 |
| Hong Kong Law Journal | 1 |
| IEEE EMBS International Conference on Biomedical and Health Informatics 2016 | 1 |
| IEEE International Conference on Healthcare Informatics 2018 | 1 |
| IEEE International Symposium on Computer-Based Medical Systems 2008 | 1 |
| Industrial and Systems Engineering Research Conference 2018 | 1 |
| Informatics for Health and Social Care | 1 |
| Information and Software Technology | 1 |
| Information Systems International Conference | 1 |
| Information Technology and Communications in Health Conference | 1 |
| Injury Prevention | 1 |
| International Conference On Computational And Bio Engineering | 1 |
| International Conference on Computer and Information Science 2022 | 1 |
| International Conference on Computer Modeling, Simulation and Algorithm 2020 | 1 |
| International Conference on e-Health Networking, Applications and Services 2016 | 1 |
| International Conference on Emerging Ubiquitous Systems and Pervasive Networks 2016 | 1 |
| International Conference on Information Quality 2010 | 1 |
| International Conference on Information Society (i-Society 2013) | 1 |
| International Conference on Information Systems | 1 |
| International Congress of the European Federation for Medical Informatics 2006 | 1 |
| International Joint Conference on Biomedical Engineering Systems and Technologies 2019 | 1 |
| International Journal of E-Health and Medical Communications | 1 |
| International Journal of Health Care Quality Assurance | 1 |
| International Journal of Healthcare Information Systems and Informatics | 1 |
| International Journal of Healthcare Management | 1 |
| International Journal of Information Management | 2 |
| International Journal of Medical Informatics | 7 |
| International Journal of Pediatric Obesity | 1 |
| International Journal of Population Data Science | 3 |
| International Journal of Social Research Methodology | 1 |
| IST-Africa Conference 2011 | 1 |
| JCO Clinical Cancer Informatics | 3 |
| Joint Conference on Knowledge-Based Software Engineering | 1 |
| Journal of Biomedical Informatics | 2 |
| Journal of Cardiothoracic and Vascular Anesthesia | 1 |
| Journal of Clinical Epidemiology | 1 |
| Journal of General Internal Medicine | 1 |
| Journal of Healthcare Engineering | 1 |
| Journal of Healthcare Informatics Research | 1 |
| Journal of Korean Medical Science | 1 |
| Journal of Medical Internet Research | 4 |
| Journal of Medical Internet Research Medical Informatics | 2 |
| Journal of Medical Systems | 2 |
| Journal of Medicine & Public Health | 1 |
| Journal of Nursing Care Quality | 1 |
| Journal of Oncology Practice | 1 |
| Journal of Public Health Management and Practice | 1 |
| Journal of the American College of Surgeons | 1 |
| Journal of the American Medical Informatics Association | 4 |
| Journal of the American Medical Informatics Association Open | 2 |
| Journal of the International AIDS Society | 1 |
| Malawi Medical Journal | 2 |
| Medical Care | 2 |
| MEDINFO 2010 | 7 |
| Neurology | 1 |
| Obstetrics & Gynecology | 1 |
| Online Journal of Public Health Informatics | 2 |
| Open Access Journal of Clinical Trials | 1 |
| Orphanet Journal of Rare Diseases | 1 |
| Pacific Asia Journal of the Association for Information Systems | 1 |
| Pediatric Critical Care Medicine | 1 |
| Pediatrics | 1 |
| Perspectives in Health Information Management | 3 |
| Pharmacy and Therapeutics | 1 |
| PLoS One | 4 |
| Policy, Politics, & Nursing Practice | 1 |
| Public Health Management Practice | 1 |
| Public Health Reports | 1 |
| Respir Care | 1 |
| SA Journal of Information Management | 1 |
| Saudi Pharmaceutical Journal | 1 |
| Scientific Reports | 1 |
| Statistical Methods in Medical Research | 1 |
| Studies in Health Technology and Informatics | 3 |
| Summit on Translational Bioinformatics | 1 |
| Systemic Practice and Action Research | 1 |
| Telemedicine and e-Health | 2 |
| The Annals of Family Medicine | 1 |
| The Conversation | 1 |
| The Lancet Digital Health | 1 |
| Topics in Health Information Management | 1 |
| Vaccine | 1 |
| Wireless Personal Communications | 1 |
| Yearbook of Medical Informatics | 1 |
