# Supplementary material for: Digital Health Data Quality Issues: Systematic Review
Source: J Med Internet Res. 2023 Mar 31;25:e42615. doi: 10.2196/42615 (PMC10131725; doi:10.2196/42615)
Supplement: Multimedia Appendix 5 [file jmir_v25i1e42615_app5.docx]

## Appendix 5: Data Quality Definitions

| DQ Definition | Reference |
| --- | --- |
| **DQ: Context Aware Perspective** | |
| The totality of features & characteristics of an entity that bears on its ability to satisfy stated and implied needs | [70] |
| Data’s “fitness for use” and can be described by a set of dimensions (e.g., accuracy and completeness) | [69] |
| The ability of the data to fulfil the purpose for which they were collected or fit for use. The concept of ‘fitness for use’ emphasises the importance of taking the end user’s perspective of quality into account because it is the end users who will decide whether a product is fit for use or is conforming to specific requirements | [31] |
| Data which is accurate, reliable, “fit for use” and relevant | [71] |
| Data fit for use, where fitness for use produces accurate, complete, and timely data accessible to stakeholders and relevant to their tasks | [72] |
| DQ is “fit-for-use” in that its determinants are dependent on the data consumer's expectations, in the context of a specific purpose for data use. | [73] |
| DQ is most commonly deﬁned as ‘ﬁtness for use’ | [20] |
| **DQ: Context Agnostic Perspective** |  |
| Documentation and contents of data within an electronic medical record (EMR) must be accurate, complete, concise, consistent and universally understood by users of the data, and must support the legal business record of the organization by maintaining the required parameters such as consistency, completeness and accuracy. | [67] |
| EHR DQ dimensions: completeness, correctness, concordance, plausibility, and currency. | [22] |
| Relevant, necessary, accurate, complete, and updated data | [74] |
| Data that are accurate, relevant, valid, reliable, legible, complete, and available when it is needed by decision-makers for healthcare delivery and planning purposes; DQ consists of six primary dimensions, which includes completeness, consistency, conformity, accuracy, integrity and timeliness | [31] |
| Variations in expected data versus collected data (e.g., timeliness, accuracy) are collectively referred to as DQ | [75] |
| Three DQ categories: conformance, completeness, and plausibility | [76] |
| EMR DQ dimensions: correctness (i.e., accuracy), completeness, concordance (i.e., accessibility), currency (i.e., timeliness), and plausibility (i.e., relevancy). | [17] |
| The core framework includes three constructs of DQ: complete, correct, and current data… EHR data completeness can be defined in multiple ways, depending upon intended use, and that, in turn, efforts to calculate rates of records completeness would vary based upon these different definitions and uses | [18] |
| “accuracy, believability, reputation, objectivity, factuality, consistency, freedom from bias, correctness, and unambiguousness.” | [21] |
| Three categories: currency, completeness, and correctness. To estimate correctness, two further categories—plausibility and concordance—were used | [54] |
| A proper assessment of DQ will examine the data from several perspectives or dimensions including validity, accuracy, completeness, relevance, timeliness, availability, comparability, consistency, duplication, integrity and conformity | [77] |
| Accuracy, availability, usability, integrity, consistency, standardisation and timeliness are some characteristics of high-quality data | [68] |
