# Supplementary material for: Digital Health Data Quality Issues: Systematic Review
Source: J Med Internet Res. 2023 Mar 31;25:e42615. doi: 10.2196/42615 (PMC10131725; doi:10.2196/42615)
Supplement: Multimedia Appendix 6 [file jmir_v25i1e42615_app6.docx]

## Appendix 6: Evidence of the Sub-Theme for Each DQ Dimension

| **Dimension** | **Sub-Theme** | **Reference** |
| --- | --- | --- |
| Accuracy | Validity | [19, 20, 38, 41, 52, 55, 80, 85, 90, 95, 112, 114, 116-125] |
|  | Correctness | [9, 11, 14, 16, 18, 21, 32, 34, 38, 39, 41, 42, 46, 47, 51, 54-56, 67, 85, 86, 88-90, 93, 95, 96, 98, 99, 193, 196, 107, 112, 113, 116, 119, 121, 123, 126, 127-129] |
|  | Integrity | [8, 10, 31, 34, 51,72, 86, 88, 92, 95, 109, 112, 114, 115, 119, 130-134] |
|  | Conformance | [19, 31, 35, 76, 85, 95, 135] |
|  | Plausibility | [14, 16, 18, 19, 32, 35, 38, 54, 57, 58, 85, 95, 101, 107, 112, 115, 135] |
|  | Veracity | [21, 86] |
|  | Accurate Diagnostic Data | [16, 34, 36, 56, 84, 86, 90, 91, 112, 120, 123, 136, 137] |
| Consistency | Inconsistent data capturing | [16, 20, 31, 36, 40, 52, 55, 59, 79, 84, 86, 87, 88, 93, 94, 99, 102, 103, 106, 108, 113, 114, 120, 121, 124, 128, 130, 136, 138-141] |
|  | Standardisation | [9, 11, 16, 34, 35, 38, 40, 44, 46, 47, 52, 57, 59, 84, 93, 99, 100, 106, 114, 117, 119, 124, 126, 128, 142-145] |
|  | Concordance | [12, 14-16, 18, 20, 38, 58, 67, 71, 80, 90, 94, 97, 107, 114, 124, 132, 140, 146, 147] |
|  | Uniqueness | [34, 36, 39, 48, 56, 87, 92, 104, 106, 109, 114, 136, 172, 173] |
|  | Data variability | [11, 34, 56, 59, 98, 101, 105, 112, 128, 144, 148-151] |
|  | Temporal variability | [38, 40, 43-45, 90, 93, 105, 121, 124, 126, 145, 152] |
|  | System differences | [34, 36, 39, 46, 48, 59, 79, 84, 88, 102, 136, 138] |
|  | Semantic consistency | [16, 20, 34, 47, 52, 55, 93, 112, 114, 121] |
|  | Structuredness | [9, 18, 20, 33, 80, 82, 112 |
|  | Representational consistency | [15, 20, 40, 47] |
| Completeness | Missing data | [10-12, 14-16, 22, 67, 31, 32, 34, 35, 38, 39, 41, 42, 44, 45 47, 52, 55, 57, 59, 60, 61, 69, 71, 79, 80, 85-90, 91, 93, 94, 95, 97-99, 102, 104, 109, 112, 122, 126, 128, 130, 131, 139, 141, 153-162] |
|  | Level of Completeness | [9, 16, 20, 32, 34, 38, 48, 53, 55, 57, 62, 68, 69, 80, 86, 94-97, 99, 100, 138, 157, 163] |
|  | Representativeness | [15, 16, 18, 20, 39, 41-43, 86, 90, 101, 165, 171] |
|  | Fragmentation | [18, 37, 88-93] |
|  | Breadth of documentation | [12, 18] |
| Contextual Validity | Contextual DQ | [8, 11, 18, 41, 78, 86, 112, 130] |
|  | Fitness for use | [20, 86, 38, 42, 54, 58, 116, 165-167] |
|  | Granularity | [16, 18, 40, 49] |
|  | Relevancy | [41, 55] |
| Accessibility | Accessibility DQ | [18, 33, 38, 39, 53, 80, 82, 126, 136, 139] |
|  | Availability | [15, 32-34, 39, 61, 113, 139] |
| Currency | Timeliness | [16, 18, 22, 31, 32, 50, 51, 54, 83, 90, 98-100, 104, 107, 110, 111, 168] |
