# Supplementary material for: Digital Health Data Quality Issues: Systematic Review
Source: J Med Internet Res. 2023 Mar 31;25:e42615. doi: 10.2196/42615 (PMC10131725; doi:10.2196/42615)
Supplement: Multimedia Appendix 7 [file jmir_v25i1e42615_app7.docx]

## Appendix 7: Evidence for the interrelationships between the dimensions of DQ

| Relationship | Evidence |
| --- | --- |
| Availability -> Currency | “Given the predominantly electronic form of communication between hospitals and general practitioners in Alberta, the inconsistency in availability of documentation in one single location can delay processes for practitioners searching for important health information.” [34] |
| Accuracy <- -> Contextual Validity (bidirectional) | “Counting complications would require interpretations of plausible temporal and causal relationships, which we were not always able to infer from observable codes. When a subject had received more than one intervention during an encounter, for example, it was difficult to determine which of the corresponding clinical events happened first and caused each other.” [16]  “We believe a lack of granularity provokes incorrectness as only part of the true clinical course of a subject can be portrayed” [16] |
| Completeness -> Accuracy | “Some providers questioned the integrity of EHR data and the potential perpetuation of errors through incomplete or repeated data entry.” [109] |
| Completeness -> Contextual validity | “In secondary use settings, EHR data completeness becomes extrinsic, and is dependent upon whether or not there are sufficient types and quantities of data to perform a research task of interest.” [42] |
| Consistency -> Accessibility | “Structured data entry (SDE) applications can prompt for completeness, provide greater accuracy and better ordering for searching and retrieval, and permit validity checks for DQ monitoring, research, and especially decision support” [80] |
| Consistency -> Contextual validity | “Information inaccuracy was also frequently observed. It was reflected as poor granularity of the diagnosis terms or disease classification codes and inadequate or non-standardized documentation of disease status or treatment details. Consequently, such information could not satisfy the information needs of a survival analysis study.” [40] |
| Consistency -> Accuracy | “We found two factors related to EHR documentation practices. False negatives and false positives in the problem list sometimes arose when the problem list was not consistently maintained and was therefore out-of-date, either because a resolved problem was not removed or because an active problem was not added (or was added after the measurement period concluded).” [90] |
| Consistency -> Completeness | “The actually corresponding procedure codes for the described operation techniques in the original study were not frequently used in our EHR, which instead employed different procedure codes; this suggests that documentation habits may have affected frequency estimates. We were unable to clearly ascertain which procedure codes represented treatment of conditions that had been documented via simultaneous diagnostic codes.”[78] |
| Consistency -> Currency | “Documentation factors: We found two factors related to EHR documentation practices. False negatives and false positives in the problem list sometimes arose when the problem list was not consistently maintained and was therefore out-of-date, either because a resolved problem was not removed or because an active problem was not added (or was added after the measurement period concluded).” [90] |
| Currency -> Accuracy | “Data is entered at different times. Some data are entered into the electronic system in real-time during admissions but other data are recorded on paper and only entered into EHR at the end of patient’s admission to the hospital. This can result in some of the data not entered into system or data recorded with errors.” [91] |
