# Supplementary material for: Digital Health Data Quality Issues: Systematic Review
Source: J Med Internet Res. 2023 Mar 31;25:e42615. doi: 10.2196/42615 (PMC10131725; doi:10.2196/42615)
Supplement: Multimedia Appendix 8 [file jmir_v25i1e42615_app8.docx]

## Appendix 8: Evidence for the outcomes of Data Quality

| Outcome | Description | Evidence |
| --- | --- | --- |
| Clinical | The extent to which digital health DQ impacts healthcare consumers. | - “Healthcare professional access to complete lifelong patient information will facilitate more effective, personalised delivery of care and increased patient safety”[63] - “When there is a gap or incomplete data from what is expected can lead to poor or delayed patient care that can lead to death, e.g., wrong results to wrong patient” [31] |
| Business process | The extent to which digital health DQ impacts the efficiency and effectiveness of healthcare-related business processes. | - The “timely and efficient access to all relevant information” [39] streamlines clinical practice and minimises unnecessary tasks [15, 63, 175] - The absence of a discharge summary can hinder communication between hospitals and general practitioners [15] |
| Clinician | The extent to which digital health DQ impacts frontline healthcare professionals. | - Nurses identified that EHR data will eliminate paperwork, improve ability to monitor patients, and decrease their workflow [33] - Poor data quality increases workload due to the documentation burden associated with inconsistent diagnosis codes [84] and inconsistency between data recorded across health settings [15] |
| Research-related | The extent to which the reusability of digital health DQ impacts clinical research outcomes. | - Well managed, high-quality digital health data facilitates data analytics [105], data retrieval [100], supporting the reusability of data [15, 16, 39, 55, 61, 90, 128] and can be applied in medical research related to clinical trials [20, 40, 42, 51, 52, 55, 57, 61, 64, 113, 166, 176-178] - The efficacy and quality of the research depend on the quality of the healthcare records [15, 20, 40, 42, 52, 58, 84, 106, 178] |
| Organisational | The extent to which digital health DQ impacts institutional finances, policy, and regulation compliance. | - “High DQ in medical records is fundamental to good clinical practice, program management and ultimately to policy decisions” [67] and further supports auditing and monitoring [40, 67, 106, 176] - DQ issues can negatively impact institutional finances and regulatory compliance. [54] |
